# Accessible Type 2 diabetes medication through stable expression of Exendin-4 in *Saccharomyces cerevisiae*

**DOI:** 10.3389/fsysb.2024.1283371

**Published:** 2024-09-02

**Authors:** Gia Balius, Kiana Imani, Zoë Petroff, Elizabeth Beer, Thiago Brasileiro Feitosa, Nathan Mccall, Lauren Paule, Neo Yixuan Peng, Joanne Shen, Vidhata Singh, Cambell Strand, Jonathan Zau, D. L. Bernick

**Affiliations:** ^1^ Department of Molecular, Cellular and Developmental Biology, University of California, Santa Cruz, CA, United States; ^2^ Department of Biomolecular Engineering and Bioinformatics, University of California, Santa Cruz, CA, United States

**Keywords:** Type 2 diabetes, Therapeutics, Medication, Exendin-4, synthetic biology, *Saccharomyces cerevisiae*, *Escherichia coli*

## Abstract

Diabetes mellitus affects roughly one in ten people globally and is the world’s ninth leading cause of death. However, a significant portion of chronic complications that contribute to mortality can be prevented with proper treatment and medication. Glucagon-like peptide 1 receptor agonists, such as Exendin-4, are one of the leading classes of Type 2 diabetes treatments but are prohibitively expensive. In this study, experimental models for recombinant Exendin-4 protein production were designed in both *Escherichia coli* and *Saccharomyces cerevisiae*. Protein expression in the chromosomally integrated *S. cerevisiae* strain was observed at the expected size of Exendin-4 and confirmed by immunoassay. This provides a foundation for the use of this Generally Regarded as Safe organism as an affordable treatment for Type 2 diabetes that can be propagated, prepared, and distributed locally.

## 1 Introduction

Type 2 diabetes (T2D) comprises around 90% of all diabetes cases and is primarily a result of insulin resistance (Goyal and Jialal, 2023). Chronic complications include heart failure, kidney disease, lower-limb amputations, blindness, and neuropathy (Kikkawa, 2000). The burden of diabetes is disproportionately borne by lower-income communities, particularly in countries that do not have medical infrastructure equipped to offer lifelong treatment (Hsu et al., 2012).

Glucagon-like peptide 1 (GLP-1) Receptor Agonists (GLP-1 RA), such as Exendin-4 (Ex-4), are one of the leading classes of T2D treatment. GLP-1 is an incretin hormone that is naturally released by intestinal L-cells in response to a rise in blood glucose levels, regulating insulin production (Holst, 2007). GLP-1s bind to human GLP-1 receptors found on pancreatic beta cells and activate adenylyl cyclase which converts ATP into cyclic adenosine monophosphate (cAMP). Increased levels of cAMP cause insulin to be released from pancreatic beta cells. Native GLP-1 is degraded by dipeptidyl peptidase-4 (DPP-4) enzymes and has an average half-life of 2 min *in vivo*.

**FIGURE 1:**
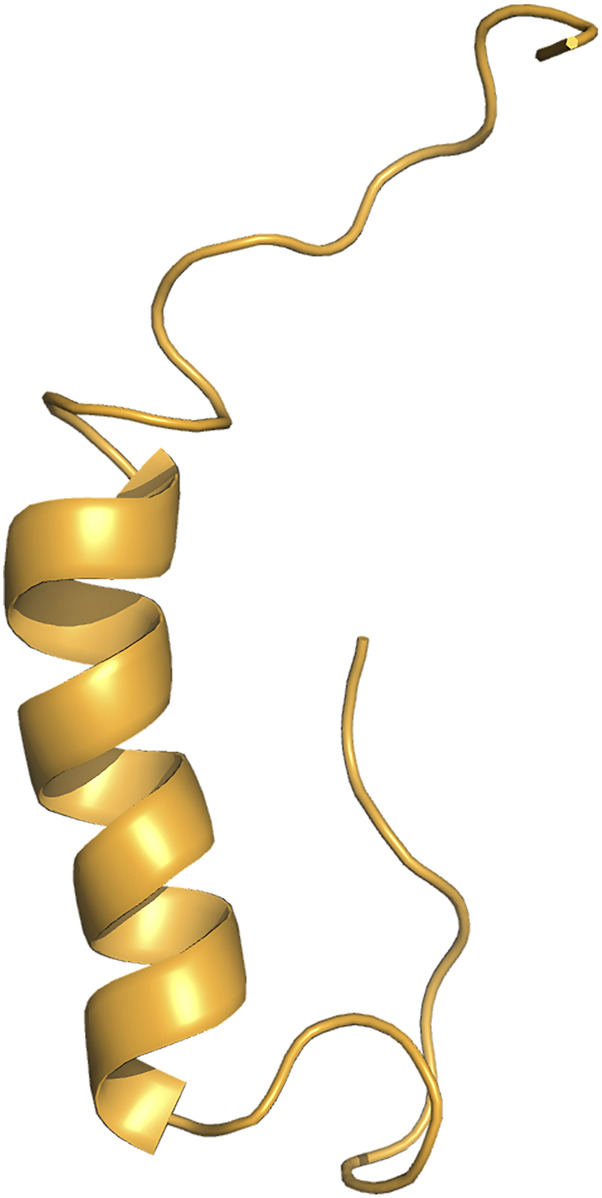
3D structure of Exendin-4. Exendin-4 (Ex-4) structure consists of a single alpha helix (Courtesy of The PyMOL Molecular Graphics System, Version 2.0 Schrödinger, LLC).

Ex-4 is a 39 amino acid peptide, (HGEGTFTSDLSKQMEEEAVRLFIEWLKNGGPSSGAPPPS) (Figure 1), originally derived from the salivary glands of the Gila Monster (*Heloderma suspectum*) (Furman, 2012). Ex-4 is resistant to degradation by DPP-4, thus resulting in a longer half-life relative to GLP-1 of around 30 min *in vivo*. The longer half-life of Ex-4 in combination with its competitive binding against native GLP-1 serves as an effective mechanism to increase both insulin production and sensitivity for T2D patients (Al-Sabah and Donnelly, 2003).

Injectable Ex-4 is currently on the market but costs upwards of $800 (USD) a month within the U.S. (Byetta #141758-74-9). The traditional method of subcutaneous injection of T2D medication can lead to a variety of safety issues and health complications (Okwen, 2011). Additionally, the price of diabetes care is rising while T2D rates are growing by a projected fifty percent in the next 30 years (Boyle et al., 2001). This is why increasing access to safe and effective diabetes treatments can broadly improve human health and particularly benefit communities suffering from the inequitable distribution of diabetes therapeutics.

Ex-4 can be produced in *Saccharomyces cerevisiae* (*S. cerevisiae*), commonly known as baker’s yeast, as a functional and cost-effective pharmaceutical treatment. As *S. cerevisiae* is Generally Regarded as Safe (GRAS) by the U.S. Food and Drug Administration (FDA) (Sewalt et al., 2016), we hypothesize bioencapsulated *S. cerevisiae* containing Ex-4 can be administered orally to bypass degradation in the stomach and avoid the need for subcutaneous injections (Kenngott et al., 2016; Kwon et al., 2013; Abid et al., 2022). Historically, *S. cerevisiae* has been employed to produce various biopharmaceutical proteins approved for human use, including GLP-1 RAs (Wang et al., 2017).

We used *Escherichia coli* (*E. coli*) in the first iteration for Ex-4 production while simultaneously pursuing *S. cerevisiae* as an edible therapeutic host. Genomic integration of the gene fragment into a GRAS organism eliminates reliance on a plasmid for protein production, allowing for Ex-4 to remain accessible in *S. cerevisiae* for many generations. With this work, we show that Ex-4 can be produced in microbial hosts as well as stably produced in *S. cerevisiae* paving the way for a safer, more affordable, and accessible T2D therapeutic.

## 2 Materials and methods

### 2.1 Exendin-4 in *Escherichia coli*

**FIGURE 2:**
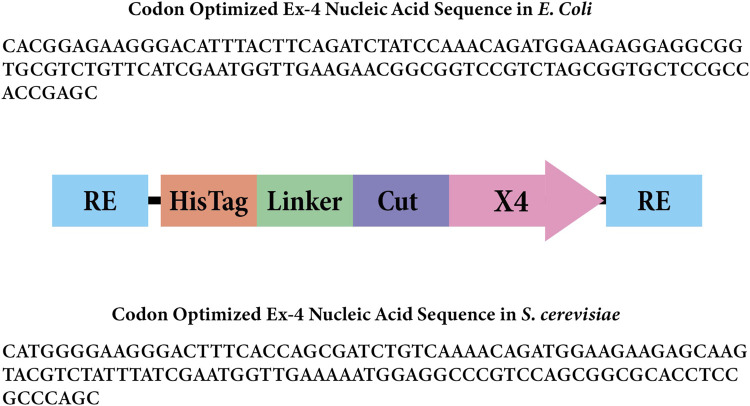
Gene insert design for Ex-4 in *Escherichia coli.* The rectangles labeled “RE” represent the BsaI restriction enzyme sites. The rectangle labeled “Linker” represents the amino acid linker, and the rectangle labeled “Cut” represents the enterokinase cut site that will remove the 6x His-tag and linker. The arrow labeled “X4” represents the codon-optimized Ex-4 insert for *Escherichia coli*.

The Ex-4 gene fragment was inserted into the pET28 expression plasmid as a replacement for the encoded GFP (AddGene #60733). Golden Gate assembly (GGA) was utilized for this construction by incorporating BsaI sites on both the insert and plasmid backbone. We incorporated a 6X-His-tag, 10 amino acid linker, and enterokinase cut site (Figure 2). The pET28 backbone was linearized using Q5 polymerase (NEB#M0491) following the manufacturer’s instructions with 10-step touchdown annealing from 70°C to 65°C. PCR products were treated with DpnI to degrade the initial template followed by heat degradation of the enzyme. The pET28:Ex4 plasmid was assembled using GGA at a 1:1 insert to backbone ratio using the NEBridge^®^ Golden Gate Assembly kit (#E1601 with BsaI-HF-v2^®^) using the NEB 50-step protocol. pET28:Ex-4 transformants were produced using *E. coli* DH5ɑ chemicompetent cells (Inoue protocol, Sambrook and Russell, 2006) using 10 ng plasmid using the heat-shock protocol described (Green and Sambrook, 2012). Cells incubated in SOC were plated on 50 μg/mL Kanamycin LB plates and incubated overnight at 37°C.

Transformant colonies were diluted in 10 µL ddH2O and with 1 µL used as a template in a OneTaq polymerase (NEB#M0480) reaction following the manufacturer’s recommendation using 10-step touchdown annealing from 69°C to 64°C, followed by 20 steps using 59°C annealing. Transformant colonies deemed successful were grown overnight using 50 μg/μL LB/Kan liquid media, and plasmids were extracted (Monarch^®^ Plasmid Miniprep Kit #T1010S). Plasmid concentration and quality were evaluated by NanoDrop™ and sequenced using Sanger Sequencing.

Successfully cloned plasmids containing our desired gene insert were then transformed again into a T7 expression host of *E. coli* BL21 (DE3). *E. coli* BL21 (DE3) LB/Kan (50 μg/mL) liquid cultures were induced at an OD of 0.6–0.8 with 0.4 mM IPTG. Cultures were incubated at 17°C overnight and shaking at 200 rpm. Samples were pelleted at 1,100 rcf for 30 min. A lysis buffer was made with 50 mM potassium phosphate at pH 7.8, 400 mM NaCl, 100 mM KCl, 10% glycerol, 0.5% Triton X-100, and 10 mM imidazole. The cell pellets were lysed using glass beads and lysis buffer in a vortex mixer for 10 min, centrifuged at 1,100 rcf for 20 min, and the soluble supernatant was filtered using a 0.45 µm filter. Ex-4 was expected to be soluble under these conditions (APExBio #A3408).

Ex-4 was isolated using immobilized metal affinity chromatography (IMAC) containing Thermo Scientific HisPur™ Cobalt Resin (#89964). Imidazole buffers were prepared as follows: Equilibration buffer 20 mM Tris/HCl, 200 mM NaCl; pH 7.5. Washing buffer 20 mM Tris/HCl, 200 mM NaCl, 5 mM imidazole; pH 7.5. Elution Buffers were prepared using 20 mM Tris/HCl and 200 mM NaCl at varying imidazole concentrations at pH 7.5. Fractions that indicated Ex-4 presence at 150 mM and 200 mM imidazole elutions were then run on a sodium dodecyl-sulfate polyacrylamide gel electrophoresis (SDS-PAGE) gel to confirm the presence of Ex-4.

### 2.2 Exendin-4 in *Saccharomyces cerevisiae*

**FIGURE 3:**
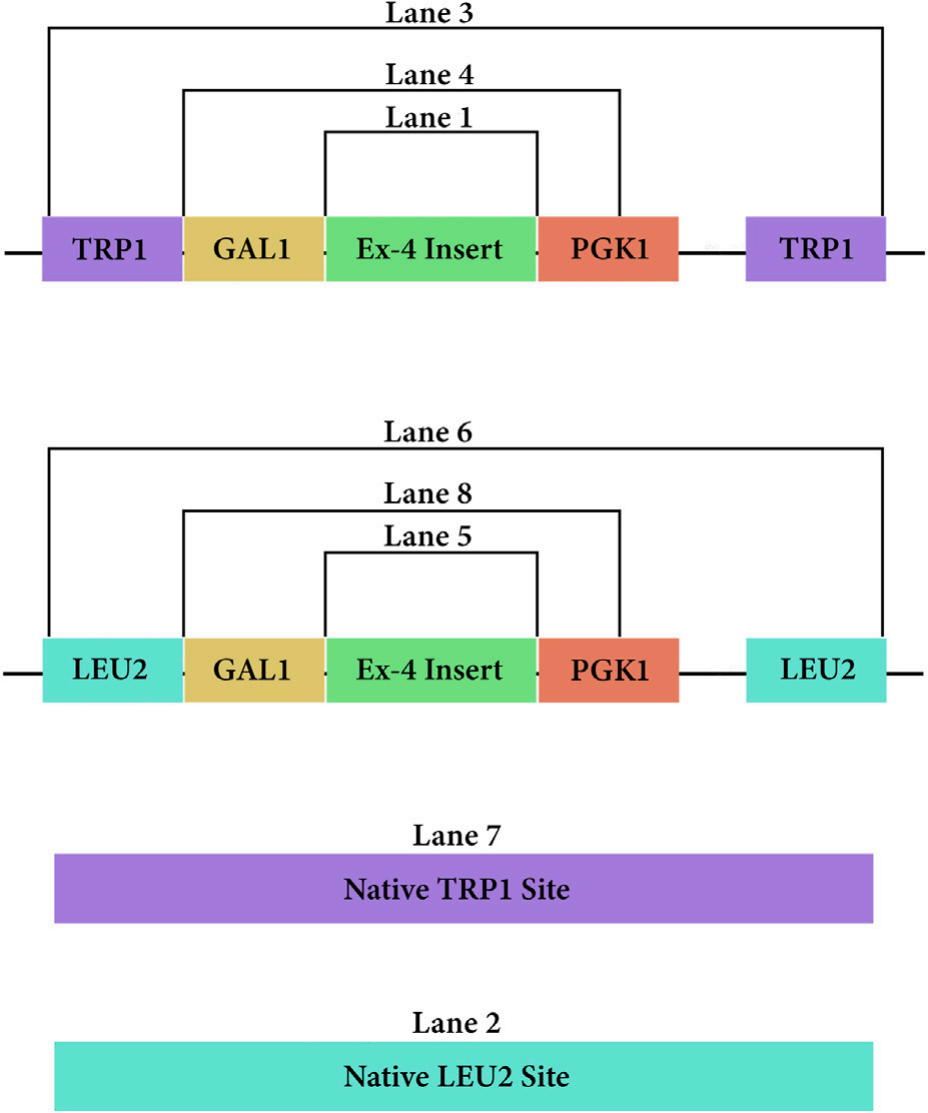
PCR amplicons of the DNA vectors from both pLEU and pTRP once integrated at their respective sites in the genome of Saccharomyces cerevisiae.

Two non-replicative fragments (pLEU and pTRP) used for chromosomal integration were prepared in *E. coli* and following the design outlined in Figure 3. Briefly, the *S. cerevisiae* Modular Cloning library (MoClo) was used to include a GAL1 promoter (Addgene#65137) and PGK1 terminator (Addgene#65161) (Lee et al., 2015) for both constructs. The codon-optimized Ex-4 sequence (Integrated DNA Technologies, 2023) includes a C-terminal half-life extender comprising a 10X repeat of VPGVG (Jung et al., 2019). The pLEU construct included a G418 selectable marker and *leu2* homology sites. The pTRP construct contained a hygromycin-selectable marker and *trp1* homology sites. The *E. coli* replicative destination vectors mg-Int-leu2-kan_1420 and mg-Int-trp1-hyg_1432-4a (iGEM parts BBa_K4190012, BBa_K4190013, https://technology.igem.org/registry), and the GAL1, PGK1 modules were mixed with 30 fmol of the respective construct at 1:1:1:1 M ratio in a 30-step Golden Gate assembly (GGA) reaction (NEB #E1601) following manufacturers recommendations.

The Golden Gate Assembly (GGA) products were transformed into chemically competent DH5ɑ *E. coli* cells for plasmid cloning following the Addgene bacterial transformation protocol (Addgene, 2017). Transformants were plated on LB agar plates containing 50 μg/mL of kanamycin antibiotic and incubated overnight at 37°C. Colonies from each plasmid construct were picked and grown overnight on a 50 μg/mL LB/Kan index plate at 37°C, then inoculated in 2xTY Kan media overnight. The ZymoPURE II Plasmid Midiprep Kit (#D4036) protocol was followed to extract the plasmid and concentration was quantified using a NanoDrop™. Constructs were evaluated by PCR to confirm proper order of Gal1 promoter, insert and PGK1 terminator. These plasmids were then combined with FastDigest NotI from Thermo Scientific (#FD0593). Two NotI enzyme binding sites are present flanking the *leu2* and *trp1* homology regions, which cut out the *E. coli* origin of replication and the Kanamycin resistance gene. The digested product was purified and concentrated with the Zymo Research DNA clean and concentrate kit (#D4003).

*Saccharomyces cerevisiae* strain ROY 6783 was transformed with the NotI digested pLEU-ex4 product and *S. cerevisiae* strain ROY 4513 was transformed with the NotI digested pTRP-ex4 product. For the pLEU-ex4 transformations, an additional linear DNA fragment was utilized in the transformation culture. This fragment contains the CRISPR-Cas9 protein coding gene and a single guide RNA targeting the *leu2* integration sites, which creates a chromosome break on the integration sites, thus increasing the integration efficiency of the pLEU-ex4 plasmid. Recombinant *S. cerevisiae* cells were prepared using the Zymo Frozen-EZ Yeast Transformation II Kit (#T2001). The outgrowth step was completed by a 2-h incubation in 1 mL Zymo YPD plus media (#Y1003), then centrifuged and resuspended in Milli-Q water before plating. The pLEU-ex4 transformants were plated on G418 sulfate YPD plates, and the pTRP-ex4 transformants were plated on Hygromycin YPD plates. A positive control of transformants was plated on YPD, and negative controls of competent cells transformed with Milli-Q water were plated on G418 sulfate or Hygromycin YPD plates. All transformants were incubated at 30°C for 48 h pLEU-ex4 and pTRP-ex4 were replica plated onto leucine or tryptophan dropout plates, respectively. We then selected the colonies on the antibiotic plates that were not present on the amino acid dropout plates and inoculated them in liquid YPG.

We isolated *S. cerevisiae* genomic DNA from both pTRP-ex4 and pLEU-ex4 transformants with the Zymo Research YeaStar Genomic DNA Kit (#D2002). For each template DNA, the forward primer on the GAL1 promoter and the reverse primer on the PKG1 terminator were used to amplify the complete sequence of the Ex-4 gene. Primer pairs designed to flank the *leu2* homology region and *trp1* homology region were also used in separate PCR reaction mixtures to amplify these regions, respectively, for both wildtype and integrants. PCR products of the chromosomal Ex-4 gene amplicon were sent for single primer extension Sanger Sequencing. The forward and reverse primers surrounding the respective integration sites were used for separate sequencing on the forward and reverse strands on each sample.

One colony of *S. cerevisiae* pLEU-ex4 transformants and one colony of *S. cerevisiae* pTRP-ex4 transformants were picked from YPD antibiotic plates and incubated overnight at 30°C in YPG. The cultures were then inoculated into a larger volume of YPG media and left to grow until their OD600 reading was around 2.5. After growing our YPG cultures, samples were pelleted at 1,100 rpm for 30 min. Cells were lysed using glass beads, IMAC purified as described above (Section 2.1) and analyzed using an SDS-PAGE gel.

IMAC purified protein from pTRP-ex4 cells (protein referred to as Ex4_Sce_) was then used to perform a sandwich ELISA assay (Abcam Exendin-4 ELISA kit #ab272192). The sample fraction from the last wash step and a 50 mM imidazole elution step were measured in comparison to the standard Ex-4. Standard Ex-4 was diluted according to the manufacturer’s protocol. In order to ensure our experimental samples were diluted to a range that was useable on the ELISA, samples were diluted in Phosphate-buffered saline to a concentration of 1.36 × 10^−2^ μg/mL, accounting for the Ex4_Sce_ mass extinction coefficient of 0.526. Further dilution occurred to 1.7 ng/mL using the provided diluent, which was then used for a six step 2-fold dilution series. Further steps of the ELISA were conducted according to the manufacturer’s instructions. The BioTek Synergy H1 microplate reader was utilized to read the absorbance of samples at 450 nm (Supplementary Figures S1, S2).

**FIGURE 4:**
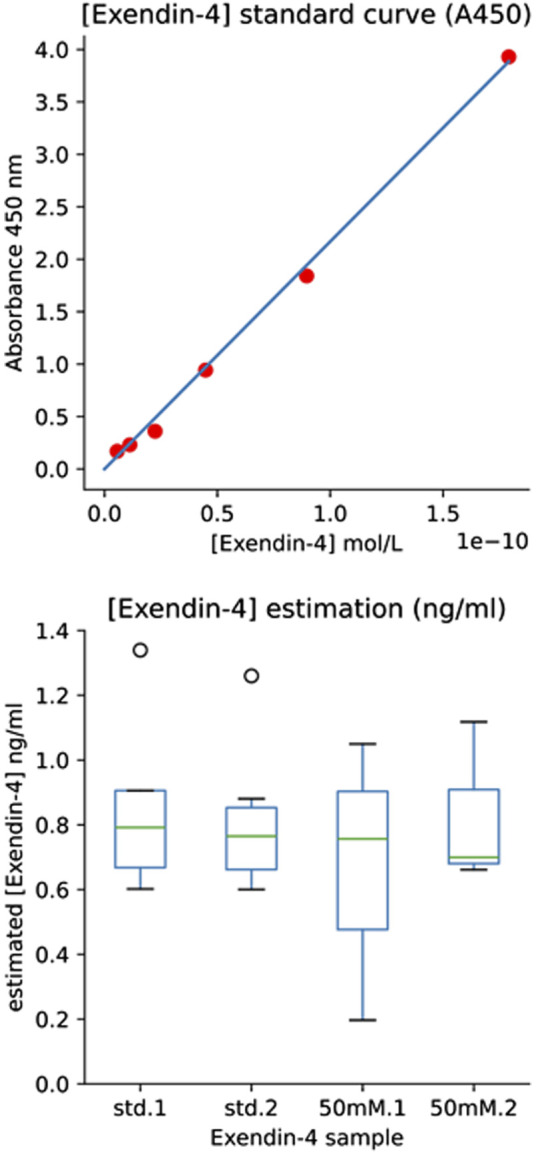
ELISA Results Providing Estimate of [Exendin-4] by Immunoassay. Top panel: [Exendin-4] Standard Curve (A450). Standard curve of A450 absorbance values for each standard concentration (y-axis) against the calculated molarity of the standard (x-axis). The six highest dilutions were plotted (1.00, 0.5, 0.25, 0.125, 0.0625, 0.03125). Bottom panel: [Exendin-4] (ng/mL) of both the manufacturers provided Exendin-4 and the 50 mM elution fraction of recombinant Ex4Sce. Std.1 and std.2 boxes refer to the loaded duplicate Ex-4 standard. 50 mM.1 and 50 mM.2 refer to the loaded duplicate of the 50 mM elution fraction of Ex4Sce.

An Ex-4 standard curve was then built to record absorbance vs. molarity (Figure 4, top panel). The average absorbance value of the blank control standards were subtracted from all absorbance values and the molarity of the initial Ex4_Sce_ samples were estimated and converted to ng/mL using the molecular weight of the Ex4_Sce_ molecule. Note, the molecular weight of the standard Ex-4 is 4,186.57 g/mol whereas Ex4_Sce_ is 10,414.49 g/mol. The least dilute 50 mM Ex4_Sce_ sample absorbance values (dilutions of 0.5, 0.25, 0.125) that were consistent with the dilution series, were taken against the standard curve to produce the concentration of target protein in each sample as seen in Figure 4, bottom panel (refer to the jupyter notebook in Supplementary Material).

## 3 Results

### 3.1 *Escherichia coli* sequencing results support intended design of expression vector

**FIGURE 5:**
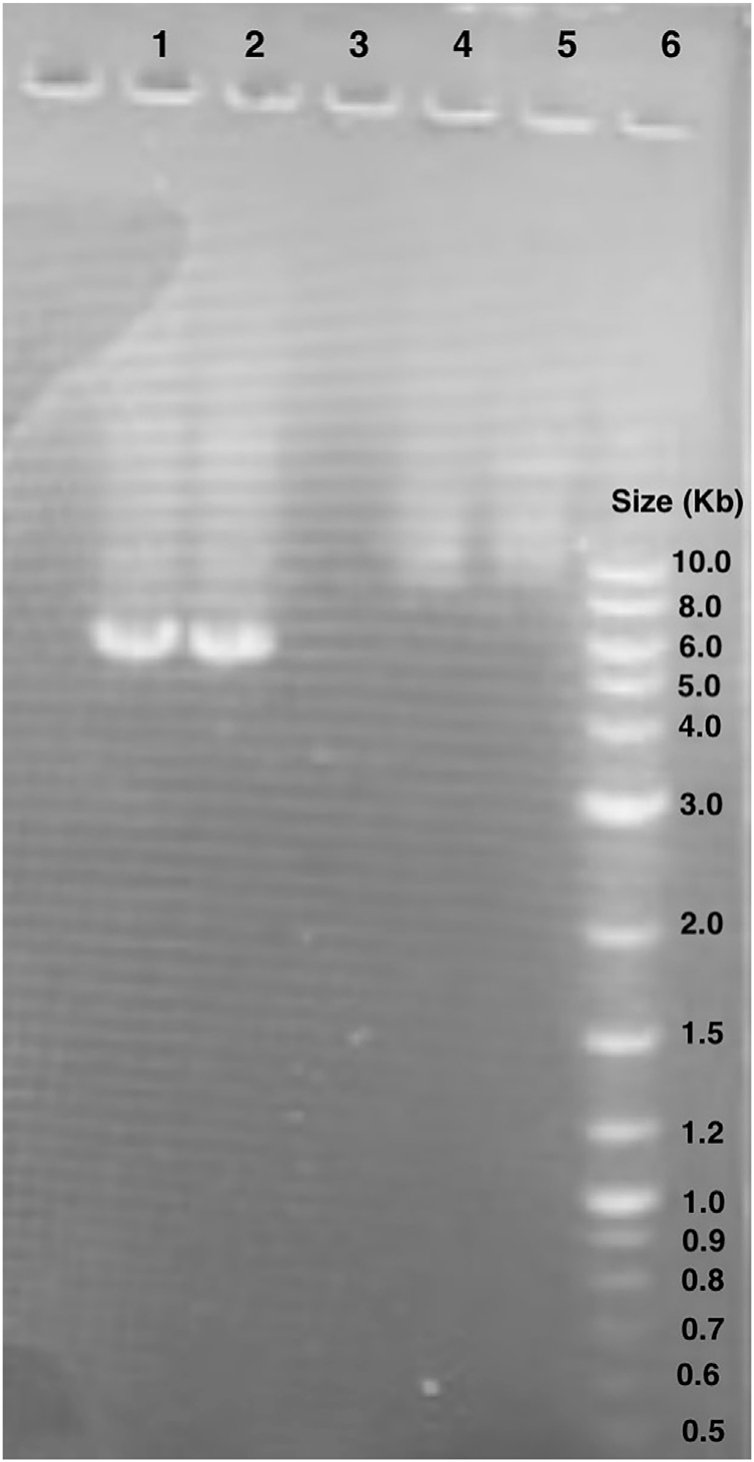
Gel electrophoresis of pET28:GFP inverse PCR reaction products. Lane 1 and 2: pET28:GFP inverse PCR products. The bands observed are around 6.0 kb. Lane 6: New England BioLabs 1 kb Plus DNA Ladder (#N3200S). Lanes 4 and 5 unrelated to this study.

We verified the linearized pET28 backbone used in GGA through gel electrophoresis (Figure 5). Bands of just below 6,000 base pairs were observed which corresponds to the expected backbone size of 5,176 base pairs, thus indicating effective primer binding and amplification.

**FIGURE 6:**
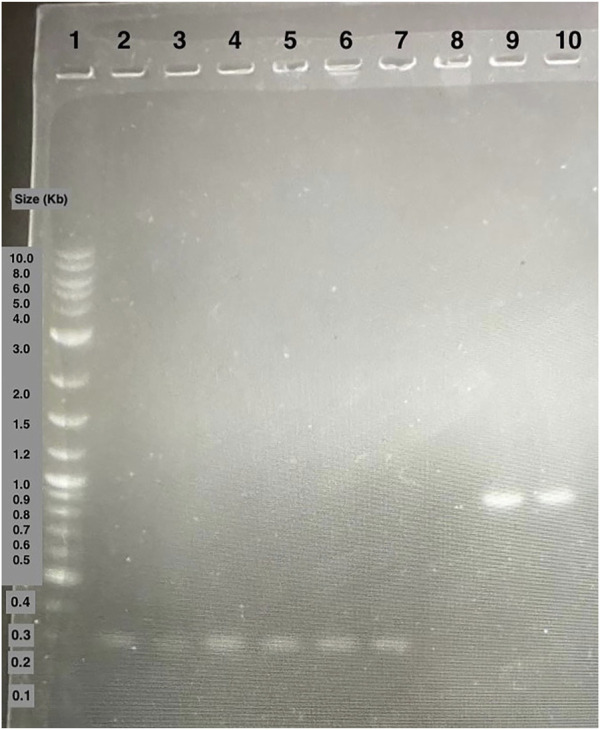
Agarose gel electrophoresis of DH5*α* colony PCR samples. Lane 1: 1 kB plus ladder. Lane 2 to 7: colony PCR samples of amplified Ex-4 gene. Lane 8 to 10: colony PCR samples of amplified GFP gene positive control.

We then introduced the Ex-4 gene into the pET28 backbone using Golden Gate Assembly (GGA). Colony PCR was then performed on transformed DH5α colonies (Figure 6). The bands present around 300 base pairs on the electrophoresis gel were observed consistent with the expected size of 347 base pairs indicating that the insert was correctly assembled within the plasmid. Colonies producing positive results (Figure 5) were sent for Sanger Sequencing. The nucleic acid chromatogram from sequencing indicated the Ex-4 gene construct was present with no mutations or deletions in the provided samples.

### 3.2 *Escherichia coli* experimental results support effective production of recombinant Ex-4

**FIGURE 7:**
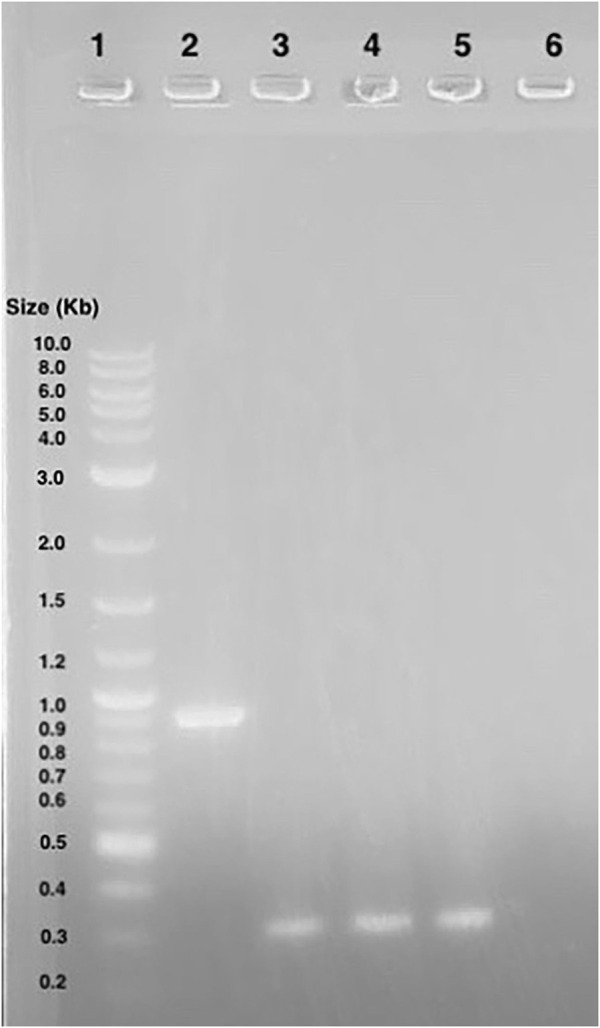
Agarose gel electrophoresis of BL21 (DE3) colony PCR samples. Lane 1: 1 kB plus ladder. Lane 2: GFP gene control amplicons. Lane 3 to 5: Ex-4 gene amplicons.

A gel of colony PCR products from BL21 (DE3) *E. coli* transformants confirmed the presence of the Ex-4 encoding plasmid. The displayed band sizes are about 300 base pairs which corresponds to the expected 347 base pair size of the Ex-4 insert (Figure 7).

**FIGURE 8:**
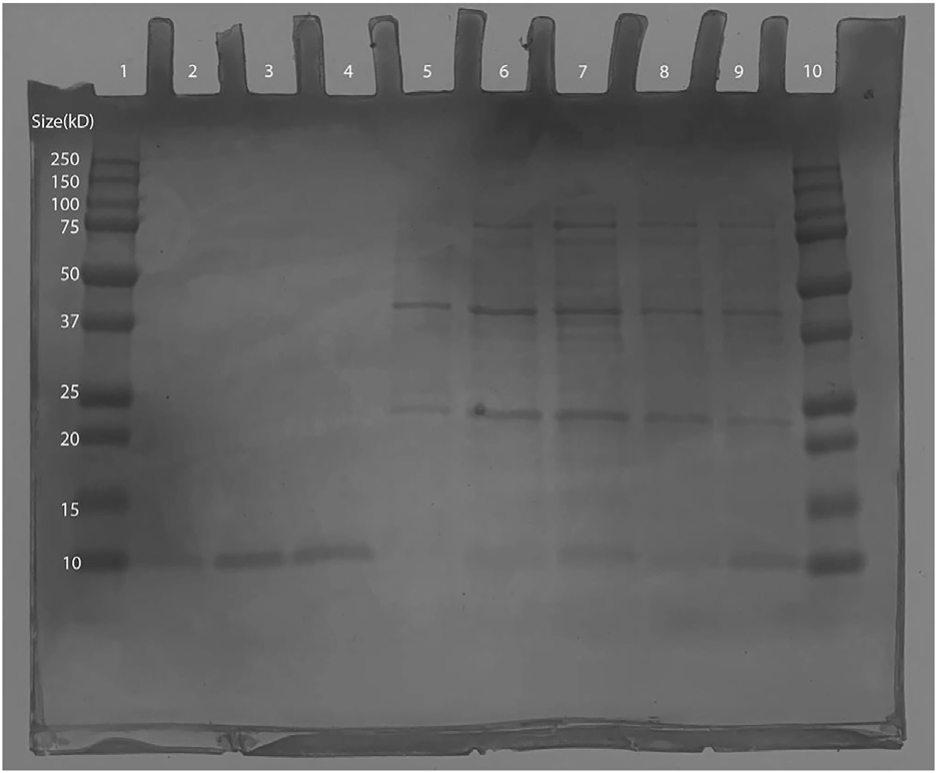
SDS-PAGE of Ex-4 in protein-producing BL21 (DE3) *Escherichia coli*. Lane 1: Bio-Rad Precision Plus Protein Dual Color Standards Ladder (#1610374). Lane 2: 100 mM Elution- 10kD band observed (expected size ∼6.45 kD). Lane 3 and 4: 150 mM elution- 10kD band observed (expected size ∼6.45 kD). Lane 5 to 7: wash samples from IMAC. Lane 10: 250 kD Bio-Rad Precision Plus Protein Dual Color Standards Ladder (#1610374).

As the expected Ex-4 insert size was shown to be within the plasmid, BL21 (DE3) transformants were induced and then purified via IMAC. Afterwards, SDS-PAGE was conducted in which bands were observed at around 10 kD: the smallest weight indicator on the protein ladder. This is close to 6.45 kD, the expected molecular weight of the His-tagged Ex-4 protein (Gasteiger et al., 2005), thus supporting successful protein purification (Figure 8). Conservatively, from a 125 mL 0.6 OD600 culture, our yields were in excess of 30 µg of protein.

### 3.3 Successful integration of Ex-4 gene cassette in *Saccharomyces cerevisiae*

**FIGURE 9:**
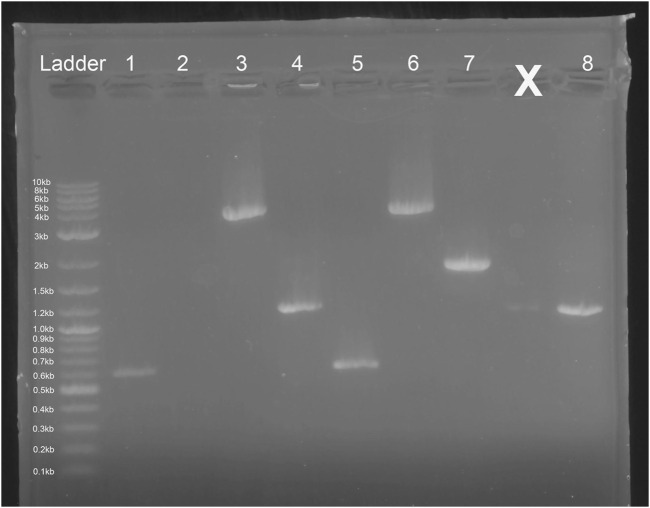
Gel Electrophoresis Results from *Saccharomyces cerevisiae* Genomic DNA Isolation. Lane 1: Ex-4 gene (expected size ∼ 603 bp) insert after pTRP-Ex4 integration. Lane 2: *leu2* site without integration (expected size ∼ 4,500 bp). Lane 3: integration of promoter, Ex-4 gene, and terminator at *trp1* site (expected size ∼ 3,500 bp). Lane 4: GAL1 promoter, Ex-4 gene, and partial terminator sequence (expected size ∼ 1,100 bp). Lane 5: Ex-4 gene insert (expected size ∼ 603 bp) after pLEU-Ex4 integration. Lane 6: integration of promoter, Ex-4 gene, and terminator at *leu2* site (expected size ∼ 4,000 bp). Lane 7: *trp1* site without integration (expected size ∼1800 bp). Lane 8: GAL1 promoter, Ex-4 gene, and partial terminator sequence (expected size ∼ 1,100 bp).

*E. coli* colony growth of GGA transformants on kanamycin selection plates indicated successful plasmid transformation. Plasmids were then linearized and transformed into competent *S. cerevisiae*. Colony growth was observed on plates of experimental transformants and positive control plates. No colonies were observed on negative control plates. Since both plasmids do not include an origin of replication for *S. cerevisiae*, colony growth indicated successful integration of the gene cassette into the chromosome. The genomic DNA gel electrophoresis showed bands at the expected nucleotide size for the respective amplicons at both the *leu2* and *trp1* sites (Figures 3, 9), confirming successful GGA, chromosomal integration, and orientation of gene blocks. To confirm these nucleotide segments matched the intended design, we sent the PCR products for Sanger Sequencing in which a 100% match was observed for each integration at each *leu2* and *trp1* site.

### 3.4 Galactose induced expression of recombinant *Saccharomyces cerevisiae* produces Ex-4 consistent protein

**FIGURE 10:**
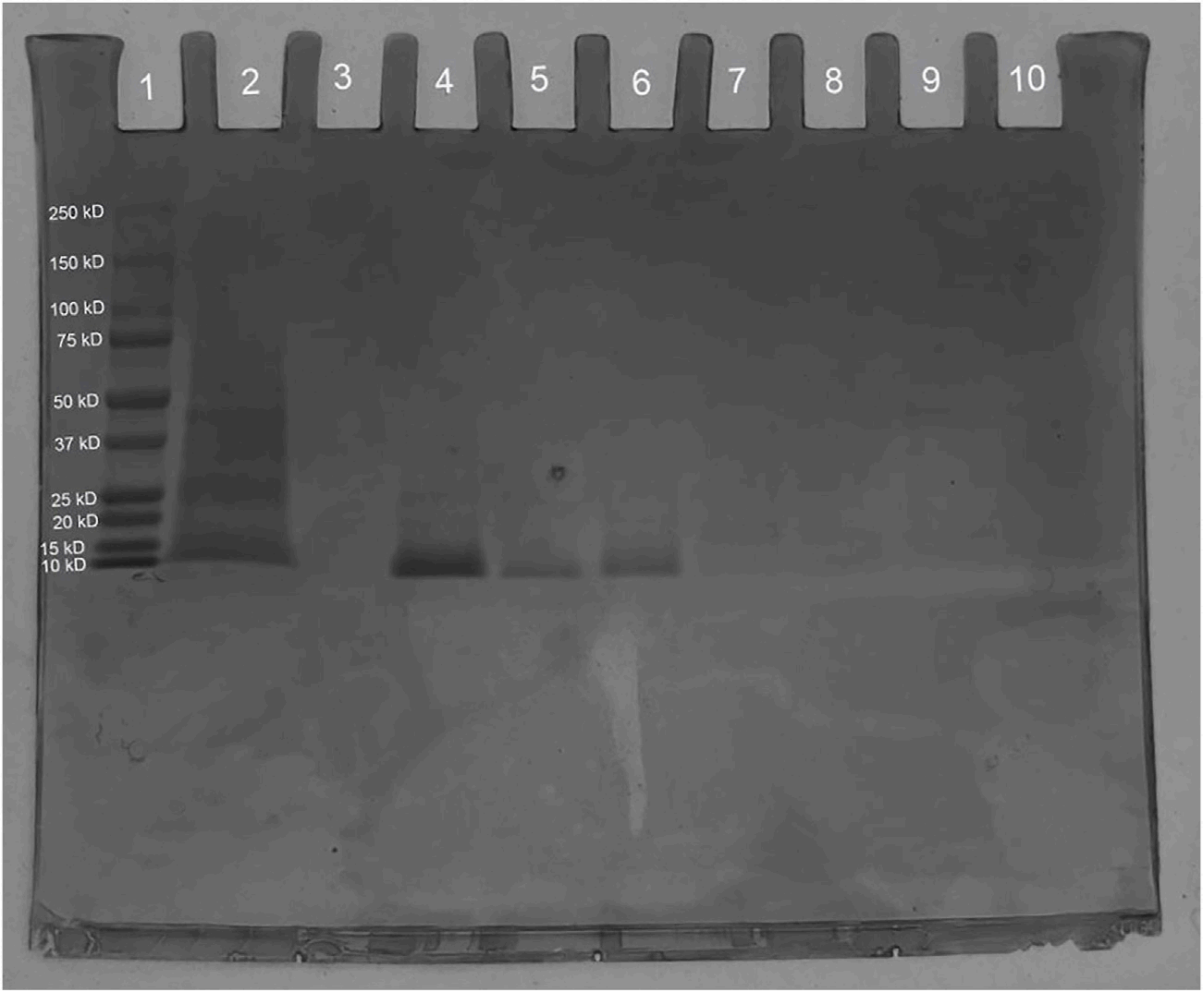
SDS-PAGE of synthesized Ex4Sce expressed from the *trp1* site. Lane 1: Bio-Rad Precision Plus Protein Dual Color Standards Ladder (#1610374), Lane 2: Unpurified cell lysis product. Lane 3: IMAC flowthrough. Lane 4: IMAC wash step. Lane 5: 50 mM imidazole elution. Lane 6: 100 mM imidazole elution.

The recombinant *S. cerevisiae* was grown in YPG for recombinant Ex-4 induction. On the SDS-PAGE, lanes 4 through 6 (Figure 10) all contain IMAC elution bands at around 10 kD, which is consistent with the expected size of the His-tagged Ex-4 protein with half-life extender (Ex4_Sce_) of 10.5 kD (Gasteiger et al., 2005). From a 5 mL 2.5 OD600 culture, our protein yields were in excess of 30 μg with significant loss in the final wash step. These observed bands indicate the success of our designed Ex-4 expression system.

### 3.5 Recombinant Ex4_Sce_ peptide is confirmed by sandwich ELISA

Ex4_Sce_ protein was bound to Ex-4 specific antibody in a sandwich ELISA (Abcam #ab272192). The initial measured mass of the Ex4_Sce_ sample was 1.7 ng/mL (see methods). Once samples were diluted for the ELISA assay, the average median (IQR) concentration estimated for the Ex-4 standard was 0.78 (0.66–0.88) ng/mL and the 50 mM elution fraction was 0.73 (0 .58–0.91) ng/mL (Figure 4, right panel). The range of the estimated concentration of Ex4_Sce_ derived from the 50 mM sample appears to be wider than what we see in the standard, suggesting variability of antibody binding of the Ex4_Sce_ sample compared to that of the standard. In comparing ELISA detectable mass to the initial measured mass, we see the median score for antibody detectable peptide is 43% (0.73/1.7 ng/mL) of the original.

## 4 Discussion

*E. coli* provided our first successful iteration of Ex-4 synthesis, which was confirmed using both plasmid sequencing and IMAC protein purification visualized with SDS-PAGE. From these results, we conclude that our gene insert design is capable of synthesizing putative Ex-4 and that our plasmid is properly assembled. Preliminary work within *E. coli* supported moving forward to a more complex and edible eukaryotic system for Ex-4 expression: *S. cerevisiae*.

We synthesized the chromosomal integrated Ex-4 gene within the *S. cerevisiae* to yield stable production of Ex-4. The band sizes observed in colony PCR demonstrated successful integration of the linearized gene construct, which was further confirmed through Sanger Sequencing. This confirmation allowed us to proceed with Ex-4 induction using the GAL1 system. The isolated protein ran on an SDS-PAGE gel indicated the presence of a protein at the appropriate size of Ex-4. The ELISA assay estimated the mass of our recombinant Ex-4 and implied a relevant structural conformation due to its ability to bind to the Ex-4 specific antibody. This further implies that a significant portion of this protein would be available in a biological context. Ex4_Sce_ protein includes both a 6x-His tag and a 10X repeat of a VPGVG half-life extender which could have interfered with antibody binding. We suggest this may have produced the variability in binding compared to the standard. Further testing is needed to validate the functionality of the recombinant Ex-4. With this success, we may now move towards the next steps of enabling *S. cerevisiae* to become an accessible and novel eukaryotic host for oral Ex-4 delivery.

Cold chain storage limits access to medication for many resource constrained communities. A method of bypassing cold chain storage would be to distribute our recombinant *S. cerevisiae* cultures as Active Dry Yeast (ADY) (Hidalgo and Brandolini, 2014). ADY has a shelf-life of around 12–18 months and does not require refrigerated storage (Kumar and Kharbikar, 2021). Hydrated *S. cerevisiae* ADY can also grow in the presence of sucrose, glucose, or maltose (Marques et al., 2016; Jansen et al., 2004), three sugars that are abundantly present in most places around the world.

To make our system more accessible, we plan to replace our GAL1 promoter for Ex-4 with a lactose promoter (LAC9) (Breunig, 1989) as well as modify the *S. cerevisiae* surface proteins (Sabu et al., 2018). As lactose is abundant in milk (Schaafsma, 2008), it is an optimal induction media that is economical and accessible (FAO and GDP, 2018). With this switch, we hypothesize our *S. cerevisiae* will express Ex-4 using milk, meaning global communities will be able to continuously produce their own T2D medication locally. Additionally, by reinforcing *S. cerevisiae*’s cell wall, prior work suggests that Ex-4 will be able to bypass the gastrointestinal barrier and target epithelial M cells for final drug release (Kenngott et al., 2016; Abid et al., 2022).

In pursuit of accessibility, we aim to foster alternative approaches for production of T2D medications that could overcome current barriers related to cost, distance, and policy. Edible microbes or plants may provide an innovative platform for such solutions. Future work will require design and testing to address immunogenicity, controlled dosage, and safety of both the platform and the delivered payload.

## Data Availability

The original contributions presented in the study are included in the article/Supplementary Materials, further inquiries can be directed to the corresponding author.
