# Supplementary material for: Accessible Type 2 diabetes medication through stable expression of Exendin-4 in Saccharomyces cerevisiae
Source: Front Syst Biol. 2024 Sep 2;4:1283371. doi: 10.3389/fsysb.2024.1283371 (PMC12342014; doi:10.3389/fsysb.2024.1283371)
Supplement: Supplementary file 1 [file DataSheet2.PDF]

Supplementary Material

Table 1. *E. Coli* Primers. Primer sequences used for inverse PCR, colony PCR, and Sanger Sequencing reactions.

Primer Name	Primer Sequence
Inverse PCR GGA IIs forward	GGTATATCTCCTTCTTAAAGTTAAAC
Inverse PCR GGA IIS reverse	TGAGATCCGGCTGCTAACAAAG
Colony PCR forward	TAGATTACCTGCATGGATG
Colony PCR reverse	ACTATAACCTGCTACCCTA
Sanger Sequencing forward	AGCCAACTCAGCTTCCTTT
Sanger Sequencing reverse	ACAATTCCCCTCTAGAAAT

Table 2. *S. cerevisiae* Primers. Primer sequences used for integrative plasmid and chromosome LEU2 and TRP1 integrative sites.

Primer Name	Primer Sequence
Integrative plasmid insert forward	GACAAGGTCTCATATGATGC
Integrative plasmid insert reverse	GTCTCAGGTCTCAGGATC
LEU2 backbone forward	TGGTAGAGCCACAAACAGCCGGTACAAGC
LEU2 backbone reverse	CTTAGTTGTGAGTCGCCAG
Ex-4 insert forward	GAAGCGATGATTTTTGATC
Ex-4 insert reverse	TTATTTTAGCGTAAAGGATGG

TRP1 forward	GTCTTGAATGAACGTATACG
TRP1 reverse	GTTAAACGGATCTCGCATTG
LEU2 forward	GAAGTGAAATGGAAAGAATTTAAG
LEU2 reverse	GAGAATCTTTATTGATGGCAC

Table 3. iGEM Parts Registry Basic Parts. Functional units of DNA uploaded to the iGEM parts registry.

Part Name	Part Number	Part Type	Description	Length (bp)
pET28:GFP	BBa_K4190000	Plasmid Backbone	Vector for gene insertion and subsequent protein expression	5993
Shine-Dalgarno Sequence	BBa_K4190001	RBS	Ribosome binding sequence to ensure genetic code is read in frame	23
6xHis-Tag (*E. coli*)	BBa_K4190002	Tag	Histidine tag attached to Ex-4 to conduct immobilized metal affinity chromatography. Codon optimized for *E. coli*	18
6xHis-Tag (*S. cerevisiae*)	BBa_K4190003	Tag	Histidine tag attached to Ex-4 to conduct immobilized metal affinity	18

			chromatography. Codon optimized for *S. cerevisiae*.	
10aa GS Linker (*E. coli*)	BBa_K4190004	Protein Domain	Links His-tag to Ex-4 protein. Codon optimized for *E. coli*	30
10aa GS Linker (*S. cerevisiae*)	BBa_K4190005	Protein Domain	Links His-tag to Ex-4 protein. Codon optimized for *S. cerevisiae*	30
Enterokinase	BBa_K4190006	Other	Cut site for eventual removal of His-tag form Ex-4	15
Ex-4 Sequence Codon Optimized for *E. coli*	BBa_K4190007	Coding Region	Protein coding region for expressing Ex-4 in *E. coli*	117
GGA Flagged Backbone Primer Forward	BBa_K4190008	Primer	Flagged primer to add BsaI site to pET28 for GGA	47
GGA Flagged Backbone Primer Reverse	BBa_K4190009	Primer	Flagged primer to add BsaI site to pET28 for GGA	43
GAL1 Promoter Plasmid	BBa_K4190010	Plasmid	MoClo part containing GAL1 promoter for GGA	2204

PGK Terminator Plasmid	BBa_K4190011	Plasmid	MoClo part containing PGK terminator for GGA	1900
mg-Int-leu2-kan_1420 Backbone	BBa_K4190012	Plasmid Backbone	Vector for gene insertion and subsequent protein expression	5609
mg-int-trp1-hyg_1432-4a Backbone	BBa_K4190013	Plasmid Backbone	Vector for gene insertion and subsequent protein expression	5412
Ex-4 Sequence Codon Optimized for *S. cerevisiae*	BBa_K4190014	Coding Region	Protein coding region for expressing Ex-4 in *S. cerevisiae*	117
EBP10 Half-life Extender	BBa_K4190015	Coding Region	Extends half life of Ex-4 *in vivo*	150
TEV Cut Site	BBa_K4190016	Other	Cut site for eventual removal of His-tag	18
GLP1-R Gene Insert Optimized for *E. coli*	BBa_K4190017	Coding Region	Protein coding region for expressing GLP-1R in *E. coli*	365

Table 4. iGEM Parts Registry Composite Parts. DNA components of two or more basic parts uploaded to the iGEM parts registry.

Part Name	Part Number	Part Type	Description	Basic Parts included	Length (bp)
Ex-4 Gene Insert for *E. coli*	BBa_K4190018	Composite	His-tagged Exendin-4 coding region to be inserted into a plasmid via GGA and expressed in *E. coli*	1. Kozak Sequence<br>2. 6xHis-Tag<br>3. 10aa GS Linker<br>4. Enterokinase Cut site<br>5. Ex-4 sequence codon optimized for *E. coli*<br>6. BsaI site	250
Ex-4 Gene Insert for *S. cerevisiae*	BBa_K4190019	Composite	His-tagged Exendin-4 coding region to be inserted into a plasmid via GGA and expressed in *S. cerevisiae*	1. 6xHis-Tag<br>2. 10aa GS Linker<br>3. Enterokinase cut site<br>4. Ex-4 sequence codon optimized for *S. cerevisiae*<br>5. EBP10 half-life extender<br>6. BsaI site	369

GLP-1R Gene Insert in *E. coli*	BBa_K4190020	Composite	His-tagged GLP-1R coding region to be inserted into a plasmid via GGA and expressed in *E. coli*. Expresses only surface binding site.	1. Kozak Sequence<br>2. 6xHis-Tag<br>3. 10aa GS Linker<br>4. TEV Cut site<br>5. GLP-1R sequence codon optimized for *E. coli*<br>6. BsaI site	505
Complete Reconstructed Plasmid (pET28:Ex4)	BBa_K4190002	Plasmid	His-tagged Exendin-4 expression system in *E. coli*	1. Kozak Sequence<br>2. 6xHis-Tag<br>3. 10aa GS Linker<br>4. Enterokinase Cut site<br>5. Ex-4 sequence codon optimized for *E. coli* pET28:GFP	5395

Complete Reconstructed Plasmid (mg-Int-leu2-kan_1420:Ex 4)	BBa_K4190002	Plasmid	His-tagged Exendin-4 expression system with integration at *S. cerevisiae* Leucine homology sites within the genome	1. 6xHis-Tag<br>2. 10aa GS Linker<br>3. Enterokinase cut site<br>4. Ex-4 sequence codon optimized for *S. cerevisiae*<br>5. EBP10 half life extender<br>6. GAL1 Promoter<br>7. PGK Terminator<br>Mg-Int-leu2-kan_1420 backbone	5699
Complete Reconstructed Plasmid (mg-int-trp1-hyg_1432-4a:Ex4)	BBa_K4190023	Plasmid	His-tagged Exendin-4 expression system with integration at *S. cerevisiae* tryptophan homology sites within the genome	1. 6xHis-Tag<br>2. 10aa GS Linker<br>3. Enterokinase cut site<br>4. Ex-4 sequence codon optimized for *S. cerevisiae*<br>5. EBP10 half-life extender<br>6. GAL1 Promoter<br>7. PGK Terminator<br>Mg-int-trp1-hyg_1432-4a backbone	5502

Complete Reconstructed Plasmid (pET28:GLP1R)	[BBa_K4190024]()	Plasmid	His-tagged GLP-1R binding site expression system in *E. coli*	1. Kozak Sequence<br>2. 6xHis-Tag<br>3. 10aa GS Linker<br>4. TEV Cut site<br>5. GLP-1R sequence codon optimized for *E. coli* pET28:GFP	5650

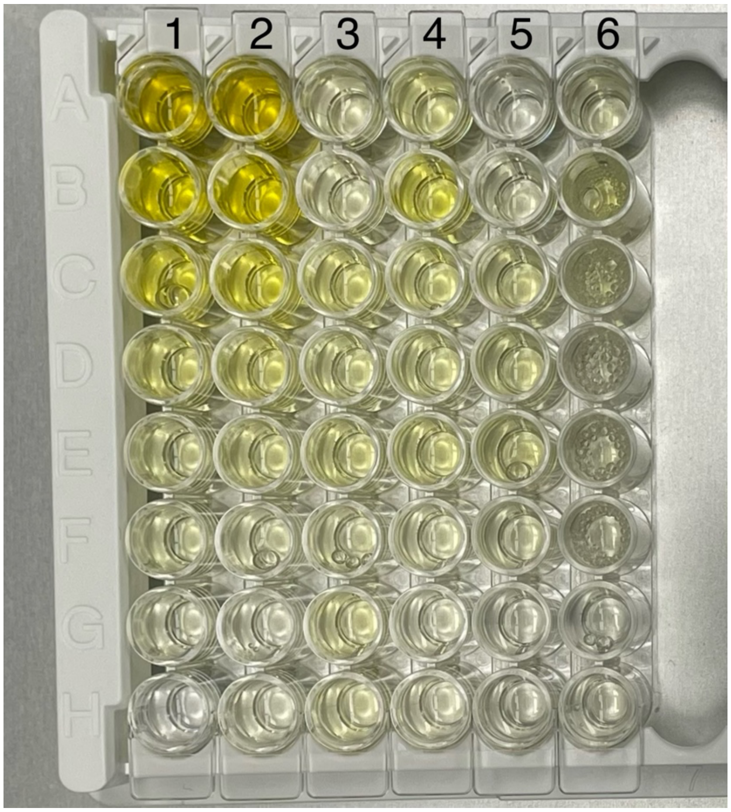

Figure 1. ELISA Microplate. Image showing ELISA microplate with wells post-assay. Column 1 & 2: Standard Ex-4 sample. Column 3 & 4: 50mM elution Ex-4 sample. Column 5 & 6: Wash Ex-4 sample. Rows A through H represent serial dilutions of samples, with the following dilution factors: A: 1.00, B: 0.5, C: 0.25, D: 0.125, E: 0.0625, F: 0.03125, G: 0.0156, H: 0.0078.

	1	2	3	4	5	6	
A	OVRFLW	3.93	0.165	0.289	0.074	0.162	450
B	1.706	1.976	0.164	0.653	0.149	1.415	450
C	1.143	0.743	0.355	0.325	0.25	0.901	450
D	0.351	0.369	0.233	0.251	0.243	0.678	450
E	0.216	0.245	0.272	0.28	0.272	0.789	450
F	0.176	0.163	0.171	0.158	0.157	0.349	450
G	0.117	1.478	0.224	0.114	0.09	0.08	450
H	0.045	0.113	0.174	0.123	0.09	0.109	450

Figure 2. ELISA A450 Absorbance. Table displaying raw absorbance readings of samples at 450nm. Samples demonstrated in same configuration as samples in Figure 1.
